# Toll-like receptor 4 in COVID-19: friend or foe?

**DOI:** 10.2217/fvl-2021-0249

**Published:** 2022-04-19

**Authors:** Suprabhat Mukherjee

**Affiliations:** ^1^Integrative Biochemistry & Immunology Laboratory, Department of Animal Science, Kazi Nazrul University, Asansol, West Bengal, 713340, India

**Keywords:** COVID-19, cytokine storm, immunotherapy, SARS-CoV-2, TLR4

## Abstract

Toll-like receptor 4, an innate immune sensor, is one of the key ‘fate-deciding’ regulators of immunity as well as COVID-19 immunopathogenesis. Suitable targeting of Toll-like receptor 4 appears to be an effective strategy to counteract the pandemic.

Since its origin from Wuhan, China, COVID-19 has taken an enormous toll on mankind. Hitherto, several intervention approaches have been aimed to combat the infectivity, transmission and various clinical manifestations associated with SARS-CoV-2 infection. Due to dearth of understanding of the mechanistic insights of the immunopathogenesis of SARS-CoV-2 infection, conception of appropriate therapeutic strategy as well as implementation of the same have achieved limited success. In this context, the molecular influence of Toll-like receptor 4 (TLR4) in COVID-19 has been deciphered as a prime regulatory factor associated with the immunity and pathogenesis of SARS-CoV-2 infection.

## Immunobiological relevance of TLR4 in COVID-19

Mortality due to COVID-19, morbidities and post recovery syndromes are majorly resulted from the disruption of immune-homeostasis in the infected individuals [1,2]. In this context, TLR4 plays the ‘fate-deciding’ role in a SARS-CoV-2-infected subject. TLR4 is a membrane-bound innate immune receptor protein (96 kDa) occurring mostly in the innate immune cells (circulating monocytes, tissue macrophages and dendritic cells) and acts as an innate immune sensor against a wide group of invading pathogens from viruses to multicellular parasites [3,4]. This pattern recognition receptor possesses three structurally and functionally distinct domains namely ligand-binding extracellular domain with leucine rich repeats, transmembrane domain and intracellular Toll/IL-1 receptor domain [3]. TLR4 possesses extreme ability to recognize different types of molecular patterns from the pathogens and activates downstream signaling pathways routed through either myeloid differentiation factor 88 (MyD88)-dependent or -independent pathways to activate transcription factors like NF-κB that govern expression of the proinflammatory cytokines [3]. Spike glycoprotein, the major infective surface protein of SARS-CoV-2 has been found as a ligand for human TLR4 recently [1] and this protein–protein interaction results in the activation of transcription factors like NF-κB, interferon regulatory factor and activator protein 1 (AP-1) encoding proinflammatory cytokines and interferons (IFNs) [5]. The *in situ* TLR4 activation in the alveolar macrophages lead to intense local inflammation resulting in accumulation of inflammatory factors that ultimately cause disruption of respiratory gas exchange, breathing problems and even death [1,6]. Such inflammatory mediators can activate different types of innate and adaptive immune cells to amplify the inflammatory milieu leading to the overt immunopathology, namely ‘cytokine storm’. Cytokine storm has direct damaging effects over the principal organs like heart, kidney, pancreas etc. [7]. In fact, cardiac inflammation due to increased expression and hyperactivation of TLR4 is a major contributing factor in heart failure in COVID patients during the infection and/or after recovery from the active infection [8]. Macrophage activation syndrome (MAS) is one of the key pathogenic hallmarks of COVID-19 that includes abundance of infiltrating macrophages and monocytes in the inflamed lung tissue resulting in the secretion of high levels of proinflammatory cytokines, IFNs and chemokines such as IFNγ, IL-6, IL-12, TGFβ, CC motif chemokine ligand 2 (CCL2), CXC motif chemokine ligand 10 (CXCL10), CXCL9 and IL-8 [9]. MAS promotes the occurrence of acute respiratory distress syndrome and limits the survivability in the affected subjects. Importantly both MAS and acute respiratory distress syndrome are regulated by TLR4. First, TLR4 activation is primarily originated through a direct interaction with the spike protein of SARS-CoV-2 that can itself cause macrophage activation and subsequent inflammatory cascade [1,9]. Second, SARS-CoV-2 infected lungs possess oxidized phospholipids formed due to oxidative stress and these oxidized phospholipids can directly activate TLR4 that in turn causes classical activation of macrophages [9]. In addition to the direct role in mediating the immunopathogenesis, TLR4 activation resulting from spike protein–TLR4 interaction also promotes the expression of angiotensin-converting enzyme 2 (ACE2) receptor expression in the alveolar cells which in turn increases the infectivity and viral load as well as hyperinflammatory events [7]. All these pro-inflammatory mediators through their endocrine action, especially TNF-α and IL-18, cause damage to cardiac, hepatic and renal tissues and most importantly increase blood pressure and hypertension-induced organ damage. Increased blood clotting (hypercoagulation) resulting from lower platelets count, alongside increased level of d-dimers formed from degradation of fibrin and activation of the mononuclear phagocytes, are the added complications in COVID-associated death cases [10]. Inflammation and thrombosis are closely related phenomena and particularly in COVID-19 patients, high levels of the pro-inflammatory cytokines have been found to be linked with hypercoagulation, activation of the platelet, migration of the leukocyte and hyperpermeability of the blood vessels [10]. Since most of the inflammatory cytokines are primarily expressed through activation of TLR4, a clear involvement of TLR4 in the COVID-associated hypercoagulation could be postulated and more attention needs to be given in this particular area for exploring new therapeutic strategies. TLR4 has also been implicated for mediating the immunopathological consequences in COVID-19 patients who have comorbidities like hyperglycaemia/diabetes, hypertension chronic kidney disease, cardiovascular diseases and other inflammatory conditions. For example, diabetic patients have a persistent level of inflammation which is due to the expression of circulating fetuin-A in the blood that activates TLR4 [11]. On the other hand, entry of SARS-CoV-2 in such individuals also causes TLR4 activation and this activation of TLR4 heightens the level of pro-inflammatory cytokines and chemokines beyond the tolerance level of the patients resulting in several pathological manifestations extending to death.

So far, only detrimental roles of TLR4 have been discussed. But, TLR4 is undoubtedly a critical innate immune sensor that selectively recognizes different invading pathogens and shapes appropriate immune response to eliminate the pathogens from the human body [3,4]. However, TLR4 occurs in several polymorphic forms (e.g., D299G, T399I) and out of these, some polymorphisms provide protection against a wide array of pathogens while others promote immunopathogenic outcomes [4]. Therefore, uncovering the ‘polymorphic culprits’ of TLR4 associated with the disruption of immune homeostasis during SARS-CoV-2 infection may usher a new hope for all.

## TLR4 as a therapeutic target for the intervention of COVID-19: recent development

COVID-19 has posed a severe challenge to the survival of mankind in terms of huge numbers of deaths, disabilities and socio-economic loss throughout the globe to date. In addition to these, the recent news on the emergence of new ‘treatment-resistant’ and ‘vaccine-escaping’ as well as highly infectious strains of SARS-CoV-2 has come out as an added concern. Repurposed drugs like broad-spectrum antiviral drug remdesivir, antiparasitic drug ivermectin, antibiotics like doxycycline and azithromycin, metabolic inhibitors like 2-deoxy-glucose etc., have been used as therapeutic options since the very beginning of the pandemic [2,8,12]. Vaccination using whole-irion vaccine, viral vector-based vaccine, mRNA-and DNA-based vaccines is currently considered as the most effective strategy in combating the pandemic. However, most of the recently reported strains of SARS-CoV-2 can bypass the effect of a number of vaccines [13]. In this direction, TLR4 could be the meaningful target for developing anti-SARS-CoV-2 drugs. Hitherto, a number of TLR4-agonists and antagonists have been either screened and/or entered in the different phases of clinical trials [14]. A number of peptides (EC-18), chemical moieties (imiquimod; phase I, hydroxychloroquine [HCQ]; phase III, artesunate) and small therapeutic molecules (PUL-042; phase III) have been found effective in targeting TLR4 and are presently undergoing through different phases of clinical trials [14]. Moreover, repurposed drugs like alogliptin, sitagliptin and linag belonging to DPP4-antagonists are also in clinical trials for their ameliorating action against TLR4-induced inflammation in diabetes COVID-19 comorbidity [15]. Similarly, fluvastatin has also been found to be effective in inhibiting TLR4-fueled cardiac inflammation in COVID infected and/or recovered patients. Apart from these, several peptide vaccines have been designed to encounter TLR4 and the subsequent inflammatory consequences [2,14]. Many benign natural products have been screened for their blocking efficacy over TLRs and these natural molecules may emerge as an effective option in the future [16]. In recent times, approval of several monoclonal antibodies for therapeutic intervention of COVID-19 has also raised the possibility of using monoclonal antibodies for blocking TLR4 activity [17]. Emergence of new strains of SARS-CoV-2 (Delta, Delta Plus and Omicron) with higher infectivity and ability of escaping the effects of the currently available vaccines has posed a new challenge to the scientific communities. In this context, TLR4 could be the option for developing host-directed immunotherapy as this receptor is conserved in humans and well known for its role in COVID-related inflammatory pathologies extending to death. Targeting TLR4 with antagonists in the severely affected patients with active infection could effectively reduce the inflammatory consequences and associated clinical manifestation. While TLR4 agonists could bolster the immunity in recipients that are at risk of infection. Taken together, it may be concluded that a clear understanding on the role of TLR4 in the immunopathology of COVID-19 and suitable targeting of this receptor with appropriate therapeutics/immunotherapeutic could pave the way to develop appropriate intervention strategy against the pandemic.
